# CXCL2 affects macrophage antitumor function and immunotherapy efficacy in esophageal squamous cell carcinoma through calcium signaling

**DOI:** 10.3389/fimmu.2026.1695387

**Published:** 2026-04-13

**Authors:** Meiying Gu, Zhenkun Liu, Xinlei Zhao, Wei He, Ruifeng Song, Tao Liu, Xiaofeng Liu, Jia Huo, Hongyi Yang, Peng Zhao, Jialei Weng, Yabing Du

**Affiliations:** 1 Department of Oncology, The First Affiliated Hospital of Zhengzhou University, Zhengzhou, Henan, China; 2 Institute for Pathology, University Hospital of Cologne, Cologne, Germany; 3 Department of Gastroenterology, The First Affiliated Hospital of Zhengzhou University, Zhengzhou, Henan, China; 4 Department of Gastrointestinal Surgery, The First Affiliated Hospital of Zhengzhou University, Zhengzhou, Henan, China; 5 Department of Gastroenterology, Anyang Yindu District Hospital of Traditional Chinese medicine, Anyang, Henan, China; 6 Department of Radiotherapy, The First Affiliated Hospital of Zhengzhou University, Zhengzhou, Henan, China; 7 Reproductive Medicine Center, The First Affiliated Hospital of Zhengzhou University, Zhengzhou, Henan, China; 8 Department of Pharmacy, The First Affiliated Hospital of Zhengzhou University, Zhengzhou, Henan, China; 9 Department of Surgical Oncology, Sir Run Run Shaw Hospital, School of Medicine, Zhejiang University, Hangzhou, Zhejiang, China

**Keywords:** calcium signaling, CXCl2, ESCC immunotherapy, macrophages, tumor microenvironment

## Abstract

**Introduction:**

Macrophages, as a major immune cell population within the tumor microenvironment (TME), play a pivotal role in disease progression and therapeutic outcomes. This study aimed to identify key macrophage subsets associated with ESCC immunotherapy response.

**Methods:**

Macrophage-specific marker gene associated with immunotherapy response was identified through single-cell RNA sequencing (scRNA-seq) analysis. The clinical relevance of CXCL2^+^ macrophages in ESCC patients was assessed by immunofluorescence staining. RNA sequencing was utilized to explore the role of CXCL2 in modulating macrophage functional phenotypes. The impact of CXCL2 on ESCC immunotherapy was validated by ESCC mouse model.

**Results:**

ScRNA-seq analysis showed that CXCL2 was predominantly expressed in macrophages within TME and significantly upregulated in immunotherapy-responsive ESCC patients. Low infiltration of intratumoral CXCL2^+^ macrophages was correlated with poor survival. Mechanistically, CXCL2 promoted the phenotypic transition of macrophages to an immune-activated state by facilitating cytosolic Ca2+ influx. Additionally, CXCL2 suppressed tumor growth and enhanced the efficacy of anti-PD-1 antibody therapy in ESCC mouse models.

**Conclusion:**

Macrophage-specific CXCL2 represents a novel biomarker for predicting immunotherapy efficacy and may potentiate the efficacy of anti-PD-1 therapy in ESCC patients.

## Introduction

Esophageal cancer ranks as the seventh most common cancer globally and the sixth leading cause of cancer-related mortality (1). In China, esophageal cancer accounts for over 50% of the global burden, with esophageal squamous cell carcinoma (ESCC) constituting approximately 95% of these cases (2, 3). Despite significant advancements in the diagnosis and treatment of ESCC, the overall 5-year survival rate remains below 20% (4). Immunotherapy, particularly programmed cell death protein 1 (PD-1) monoclonal antibodies, has shown remarkable progress in treating various malignancies (5). However, the tumor heterogeneity of ESCC and the development of drug resistance limit its therapeutic efficacy. Therefore, identifying predictive biomarkers for immunotherapeutic response and developing effective combination strategies to enhance immunotherapy efficacy are critical for improving clinical outcomes.

As a major cellular component of tumor microenvironment(TME), macrophages play a pivotal role in cancer development and progression. Tumor-associated macrophages (TAMs) represent a plastic and heterogeneous population of immune cells (6). The TME drives the differentiation of TAMs into classically activated M1 (proinflammatory) and alternatively activated M2 (anti-inflammatory) macrophages (7). M1-type macrophages secrete cytokines such as IL-12 and TNF-α to exert tumoricidal effects, whereas M2-type macrophages inhibit T-cell function by secreting transforming growth factor-β (TGF-β), IL-10, arginase 1, and other factors that promote tumor growth, metastasis, and angiogenesis (8, 9). Within the TME, macrophages predominantly exhibit an M2-like phenotype. Therefore, promoting the polarization of TAMs toward M1-like phenotype to remodel the microenvironment and enhance the efficacy of immunotherapy represents a key area of current research (10). Identifying specific macrophage subpopulations within the TME that are associated with immunotherapy response, followed by reprogramming or repolarizing TAMs to exhibit anti-tumor properties, may enhance immunotherapy efficacy.

Chemokines mediate many biological behaviors, including chemotaxis, angiogenesis and tumor growth (11). As members of the chemokine family, CXCL chemokines play crucial roles in recruiting and mobilizing immune cells, as well as regulating pathological tumor behaviors, including proliferation, invasion, and metastasis. Numerous studies have highlighted the potential of CXCLs as biomarkers, therapeutic targets, and prognostic indicators in various cancers (12). CXC chemokine ligand 2 (CXCL2) is primarily produced by tumor cells, neutrophils, and macrophages and has been shown to play a dual role in the bidirectional crosstalk between tumor cells and macrophages across diverse tumor microenvironments (13–15). Colorectal cancer cells expressing METTL3 promote CXCL2 secretion through the NF-κB pathway, which subsequently recruits M2-type macrophages and facilitates pulmonary metastasis of colorectal cancer (16). Conversely, the combination of CXCL2 plasmid DNA with inactivated Sendai virus (hemagglutinating virus of Japan) envelope (HVJ-E) suppressed the growth and lung metastasis of breast cancer by enhancing cytotoxic T-lymphocyte activity and improving the efficacy of anti-PD-1 antibodies (17). However, the expression patterns and functional roles of CXCL2 in the tumor immune microenvironment of ESCC remain unexplored.

In this study, using single-cell RNA sequencing (scRNA-seq), we demonstrated that CXCL2 was predominantly expressed in macrophages and that ESCC patients response to anti-PD-1 therapy exhibited elevated levels of CXCL2 in macrophages. Double-labeled immunofluorescence staining of our patient cohort revealed that ESCC patients with high infiltration of CD68^+^CXCL2^+^ macrophages had improved survival outcomes. CXCL2 could promote polarization of macrophages toward an anti-tumor phenotype. Mechanistically, transcriptome sequencing analysis indicated that CXCL2 recombinant protein activated calcium signaling in macrophages. Furthermore, CXCL2 enhanced the responsiveness of ESCC to anti-PD-1 therapy *in vivo*. In summary, our study elucidates the role of CXCL2 in macrophages in ESCC and highlights its potential clinical implications.

## Methods

### Single-cell RNA sequencing analysis

ScRNA-seq data from ESCC patient cohorts (GSE203115 and GSE160269) were obtained from the Gene Expression Omnibus (GEO) database. The GSE203115 dataset comprises tumor samples from ESCC patients treated with neoadjuvant chemotherapy and immunotherapy (NACI), including responders (n=2) and non-responders (n=1) (18). The GSE160269 dataset includes 60 ESCC tumor samples. Quality control, data normalization, variable gene identification, and dimensionality reduction clustering were performed using the Seurat R package (19). The identification of cell types was facilitated by the utilization of canonical markers during the process of cluster annotation. In more detail, the following genes were identified: DCN, COL14A1, COL1A2, COL1A1 for fibroblasts; TPSB2, TPSAB1, CPA3 for mast cells; VWF, PECAM1, ENG for endothelial cells; CD68, CD14 for myeloid cells; CD3E, CD3D, CD2 for T cells; CD19, CD79A, MS4A1 for B cells; KRT13, KRT5, EPCAM for epithelial cells; IGKC, IGHG1 for plasma cells; ACTA2, MYL9, MYLK for smooth muscle and pericyte cells. Concurrently, the marker genes for each cluster were identified using the FindMarkers function with the Wilcoxon rank-sum test algorithm (18). Expression levels of canonical markers were found to be significantly elevated in clusters of the same cell type. Uniform Manifold Approximation and Projection (UMAP) was employed for dimensionality reduction, resulting in the identification of 19 distinct clusters. Differentially expressed genes (DEGs) for each cluster were identified using the FindAllMarkers function. Cell types were annotated by integrating previously reported marker genes and the most significantly expressed genes. Functional enrichment analysis, including Gene Ontology (GO) and Kyoto Encyclopedia of Genes and Genomes (KEGG) pathway analyses, was conducted using the DAVID online tool.

### Double-labeling immunofluorescence assay

A human tissue microarray containing 74 ESCC tissues was purchased from Superbiotek (Shanghai, China). The clinicopathological characteristics of the patients were summarized in online **Supplementary Table 1**. Double-labeled immunofluorescence staining was performed using the TSA Fluorescence Kit. The following primary antibodies were used: anti-CD68 antibody (Cat. ab213363, Abcam) and anti-CXCL2 antibody (Cat. 26791-1-AP, Proteintech). Stained tissues were scanned and analyzed using CaseViewer software. The population density of CXCL2^+^ or CD68^+^CXCL2^+^ macrophages was quantified as the average number of positive cells in randomly selected fields of view under a 40× objective lens. ESCC patients were stratified into high and low CD68^+^CXCL2^+^ macrophage groups based on the median value as the threshold.

### Cell lines and cell culture

The murine ESCC cell line AKR was obtained from Ya Ji Bio-Tech Ltd and cultured in RPMI-1640 medium supplemented with 10% fetal bovine serum (FBS), 100 mg/mLpenicillin and streptomycin at 37°C. Bone marrow-derived macrophages (BMDMs) were isolated from the bilateral femurs and tibiae of 6-week-old C57BL/6 mice. These cells were cultured in DMEM medium containing 10% FBS, and 20 ng/ml macrophage colony-stimulating factor (M-CSF) for 5 to 7 days or were treated with 10 ng/ml recombinant CXCL2 protein for 12 hours.

### RNA extraction and quantitative real-time PCR

Total RNA was isolated from BMDMs using the RNAiso Plus reagent (Takara, Japan). RNA concentration was measured using the Qubit RNA HS Assay Kit (Thermo Fisher Scientific, USA), and cDNA synthesis was performed with the PrimeScript RT reagent kit (Takara, Japan) according to the manufacturer’s instructions. qRT-PCR was conducted using SYBR Premix ExTaq (Yeasen, Shanghai, China) on an ABI Prism 7500 Sequence Detection System (Applied Biosystems, USA). Each reaction was performed in triplicate with a final volume of 10 µl, following the manufacturer’s protocol. GAPDH was used as an internal control, and the relative mRNA expression levels of target genes were calculated using the 2^ΔΔCt^ method. Primer sequences are listed in **Supplementary Table 2**.

### Transcriptome sequencing and gene enrichment analysis

Cellular RNA was extracted as described above, and a minimum of 2×10^6^ cells per sample was ensured. RNA concentration and purity were measured using a Nanodrop spectrophotometer. High-quality RNA was quantified, and sequencing libraries were constructed and sequenced on the HiSeq X platform (Illumina, USA). Raw sequencing data were subjected to quality control using FastQC, and differential gene expression analysis was performed using the DESeq2 R package (*P* < 0.05). The results were visualized using the Ouyi Cloud platform.

### Flow cytometry

Cultured cells were harvested using trypsin, and tumor tissues from mouse models were digested with collagenase and trypsin at 37 °C for 30 minutes. Single-cell suspensions were filtered through a 70 µm filter and stained with the following antibodies for flow cytometry: Fluo-3AM (Cat. HY-D0716; MCE) or PE/Dazzle 594 anti-mouse major histocompatibility complex (MHC)-II antibody (Cat. 107648; BioLegend). Stained cells were analyzed on a BD FACSAria III flow cytometer, and data was processed using FlowJo software.

### Animal experiments

The male C57BL/6 mouse used in this experiment were purchased from SPF (Beijing) Biotechnology Co., Ltd and acclimatized to the environment for 3–5 days. AKR cells were resuspended in 100 µL of PBS at a density of 2×10^6^ cells and subcutaneously injected into the right axilla of each mouse. Once tumors became palpable (approximately 5–7 days post-injection), the mice were randomly divided into groups (5 mice per group) and treated as follows: intraperitoneal injection of 100 µg CXCL2 recombinant protein (Cat. HY-P7258; MCE) once every two days, 150 µg anti-PD-1 antibody (Cat. BP0273; BioX Cell) once every three days (20), or the combination of both. Tumor volume was measured using vernier calipers and calculated using the formula: 0.5×length×width^2^. After 15 days, the mice were sacrificed, the tumors were removed for Flow cytometry analysis. All animal experiments were conducted in accordance with the guidelines for the care and use of laboratory animals and were approved by the Animal Care and Ethics Committee of the Henan Animal Experiment Center.

### Statistical analysis

Continuous variables were presented as mean ± standard deviation (SD) and analyzed using Student’s t-test or one-way analysis of variance (ANOVA). Categorical variables were expressed as *n* (%) and compared using Pearson’s χ² test or Fisher’s exact test. Survival curves were plotted using Kaplan-Meier method with between-group comparisons assessed by the log-rank test. Cox regression models were constructed through stepwise selection with hazard ratios (HRs) reported alongside 95% confidence intervals. All statistical analyses were performed using SPSS version 25.0 and R 4.0.0 software. A two-tailed *P* value < 0.05 was considered statistically significant.

## Results

### ScRNA-seq analysis identified macrophage-specific genes associated with immunotherapy response in ESCC

**Figure 1:**
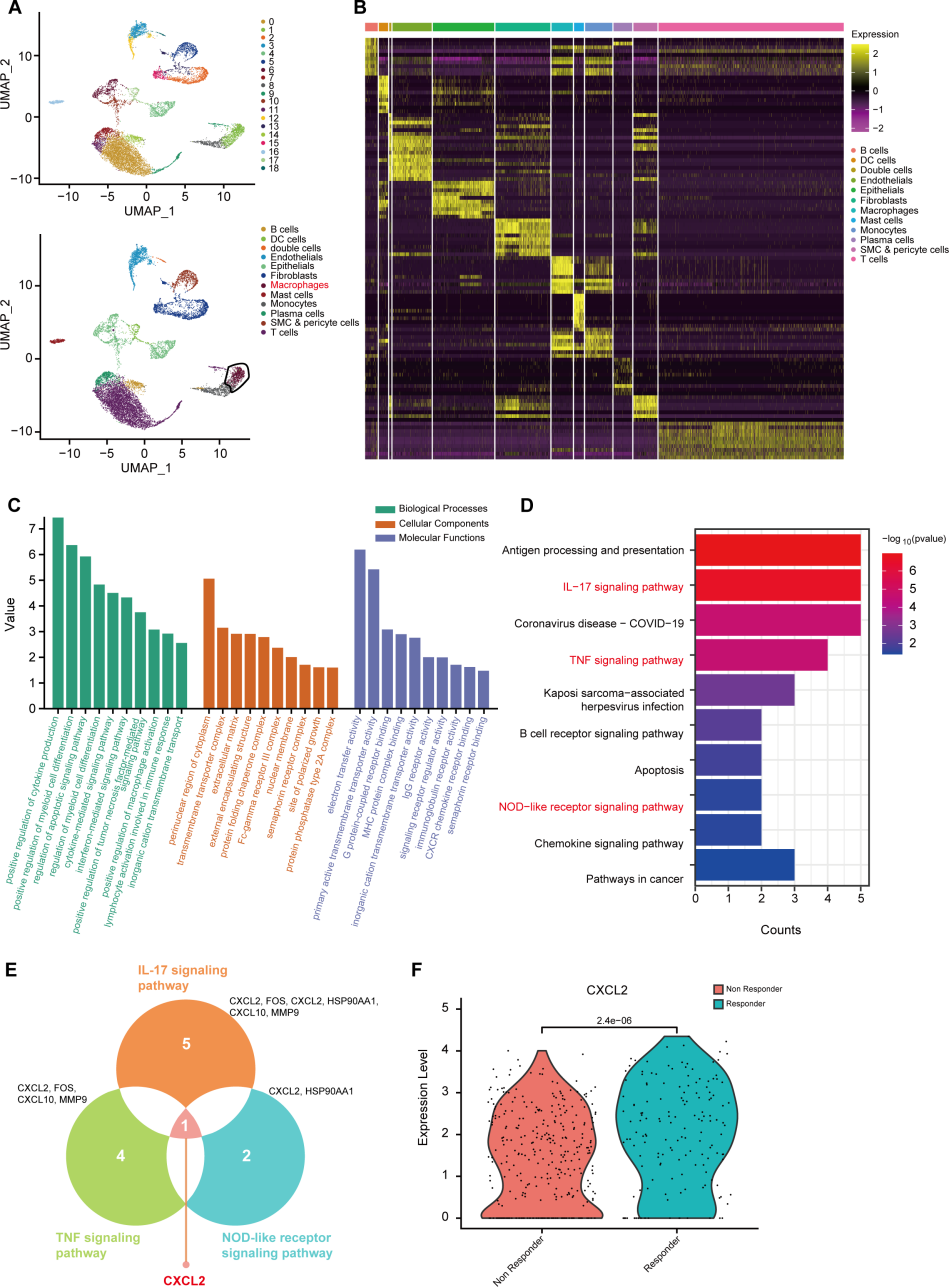
ScRNA-seq analysis identified macrophage-specific genes associated with immunotherapy response in ESCC. **(A)** UMAP plot of single cells from patients with ESCC in GSE203115 cohort. **(B)** Heatmap of marker genes in each single cell subcluster based on the clustering analysis. **(C, D)** GO and KEGG analyses of differentially expressed genes in macrophages between the responsive and non-responsive groups. **(E)**Venn diagram of intersected gene in the indicated three signaling pathways. **(F)** CXCL2 expression levels on macrophages in the responding and non-responding groups. ESCC, esophageal squamous cell carcinoma; CXCL2, CXC chemokine ligand 2; GO, gene ontology; KEGG, Kyoto Encyclopedia of Genes and Genomes; UMAP, Uniform Manifold Approximation and Projection; scRNA-seq, single-cell RNA sequencing.

To investigate key macrophage subset in the ESCC microenvironment associated with immunotherapy response, we analyzed scRNA-seq data (GSE203115) from a publicly available dataset of ESCC patients. The dataset was stratified into responsive and non-responsive groups based on their response to immunotherapy. Following principal component analysis (PCA) and uniform manifold approximation and projection (UMAP) dimensionality reduction, we identified 19 distinct cell clusters, which were further annotated into 12 cell types based on canonical marker genes, including T cell, endothelial cell, epithelial cell, fibroblast, B cell, plasma cell, and macrophage, among others (**Figure 1A**). Clustering analysis revealed significant transcriptional heterogeneity among these subpopulations (**Figure 1B**). Subsequently, we extracted differentially expressed genes in macrophages between the responsive and non-responsive groups and performed functional enrichment analysis. The gene Ontology (GO) demonstrated that these genes were mainly involved in immunoregulatory processes such as regulation of myeloid cell differentiation and cytokine-mediated signaling pathway (**Figure 1C**), while KEGG pathway analysis highlighted their enrichment in immune-related signaling pathways, including TNF, IL-17and NOD-like receptor signaling pathways (**Figure 1D**). Given the established roles of these pathways in tumor immunity (21–23), we intersected the differentially expressed genes enriched in these pathways and identified CXCL2 as the sole gene common to all three (**Figure 1E**). Further investigation revealed that CXCL2 was predominantly expressed by monocyte-derived macrophages within the ESCC (**Supplementary Figures 1A, B**). This finding was validated in an independent cohort (GSE160269) comprising 60 patients (**Supplementary Figures 1C–E**). Further investigation on the GSE203115 cohort showed that CXCL2 expression was increased in macrophages of immunotherapy-responsive ESCC patients compared to non-responsive individuals (**Figure 1F**). Collectively, our results highlight a CXCL2^+^ macrophage subpopulation linked to immunotherapy response in ESCC patients.

### High infiltration of CXCL2^+^ macrophages is positively associated with favorable prognosis in ESCC patients

**Figure 2:**
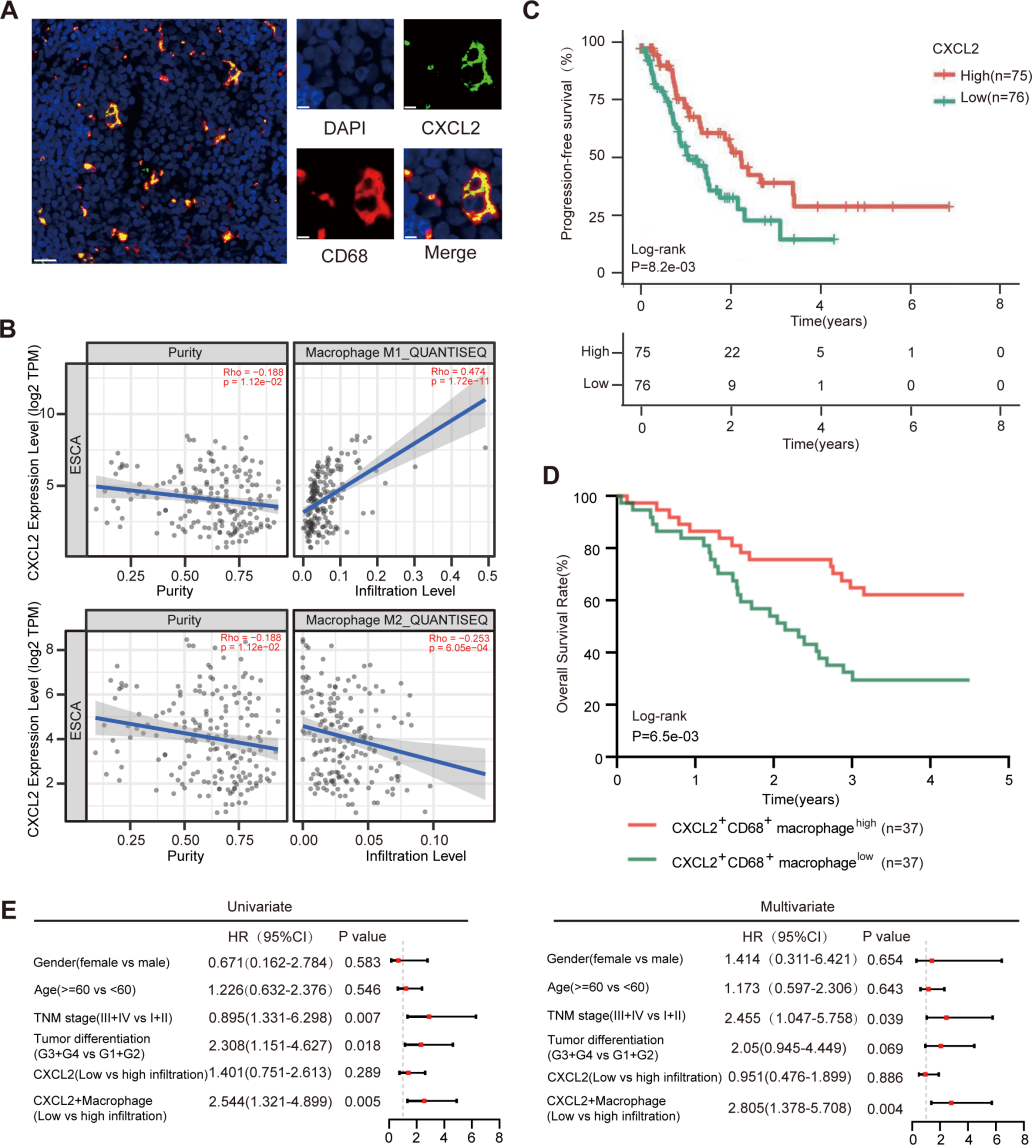
High infiltration of CXCL2^+^ macrophages is positively associated with favorable prognosis in ESCC patients. **(A)** Representative images of immunofluorescence co-staining of CD68 (red) and CXCL2 (green) in ESCC tissues. Scale bar, 20µm (left) and 5µm (right). **(B)** Pearson correlation analysis of CXCL2 expression level with the infiltration proportion of M1 or M2 macrophage in ESCC. **(C)** Kaplan-Meier curve for PFS of patients with low or high CXCL2 expression in TCGA cohort. Log-rank test. **(D)** Kaplan-Meier curve for OS of patients with low or high CXCL2^+^ macrophage population in our ESCC patient cohort. Log-rank test. **(E)** Forest plot illustrating the univariate and multivariate Cox proportional hazards regression models for OS in ESCC patients from our own cohort. ESCC, esophageal squamous cell carcinoma; CXCL2, CXC chemokine ligand 2; OS, overall survival; PFS, Progression-free survival; TNM, tumor-node-metastasis; HR, hazard ratio; CI, confidence interval.

Based on the scRNA-seq results, we performed double-labeled immunofluorescence staining of tumor tissues from ESCC patients and observed co-localization of CXCL2 and the macrophage-specific marker CD68 in ESCC tissues (**Figure 2A**; **Supplementary Figure 4**), confirming the presence of CXCL2^+^ macrophage populations in the ESCC microenvironment. Using the QUANTISEQ algorithm, we found that CXCL2 expression levels were positively correlated with M1 macrophage infiltration (R = 0.474), negatively correlated with M2 macrophage infiltration in the TCGA-ESCA cohort (R = 0.253, **Figure 2B**), and positively correlated with CD8^+^ T cell infiltration (R = 0.16, **Supplementary Figure 4**). Next, we investigated the prognostic significance of CXCL2^+^ macrophages in ESCC patients. Analysis of the TCGA cohort revealed that patients with high CXCL2 expression levels had better progression free survival (PFS) compared to those with low CXCL2 expression levels (*P* = 0.0082; **Figure 2C**). Further analysis of tissue microarrays from ESCC patients, stratified by median CXCL2^+^ macrophage infiltration levels, showed that patients with high CXCL2^+^ macrophage infiltration had significantly improved overall survival (OS) compared to those with low infiltration (*P* = 0.0065, **Figure 2D**). Univariate (HR = 2.544; 95% CI, 1.321-4.899; *P* = 0.005) and multivariate (HR = 2.805; 95% CI, 1.378-5.708; *P* = 0.004) Cox proportional hazards regression models indicated that low infiltration of CXCL2^+^ macrophages was an independent prognostic risk factor for OS in our ESCC cohort (**Figure 2E**). Overall, high infiltration of CXCL2^+^ macrophages in the TME was significantly associated with favorable ESCC patient prognosis. In addition, to verify the correlation between CXCL2 and immune response in esophageal cancer, we performed cross-cancer validation using scRNA-seq datasets from GSE207422 (15 lung cancer patients receiving immunotherapy) and GSE205506 (10 colorectal cancer patients receiving PD-1 therapy). As shown in **Supplementary Figure 3**, high CXCL2 expression within tumor-associated macrophages correlates with favorable responses to tumor immunotherapy. Furthermore, we collected tumor tissues from 17 ESCC patients undergoing immunotherapy and performed immunofluorescence staining. Patients with higher CXCL2^+^ macrophage infiltration demonstrated superior immunotherapy response (**Supplementary Figure 6**).

### CXCL2 regulated the transition of macrophages to an immune-activated state by mediating cytoplasmic calcium influx

**Figure 3:**
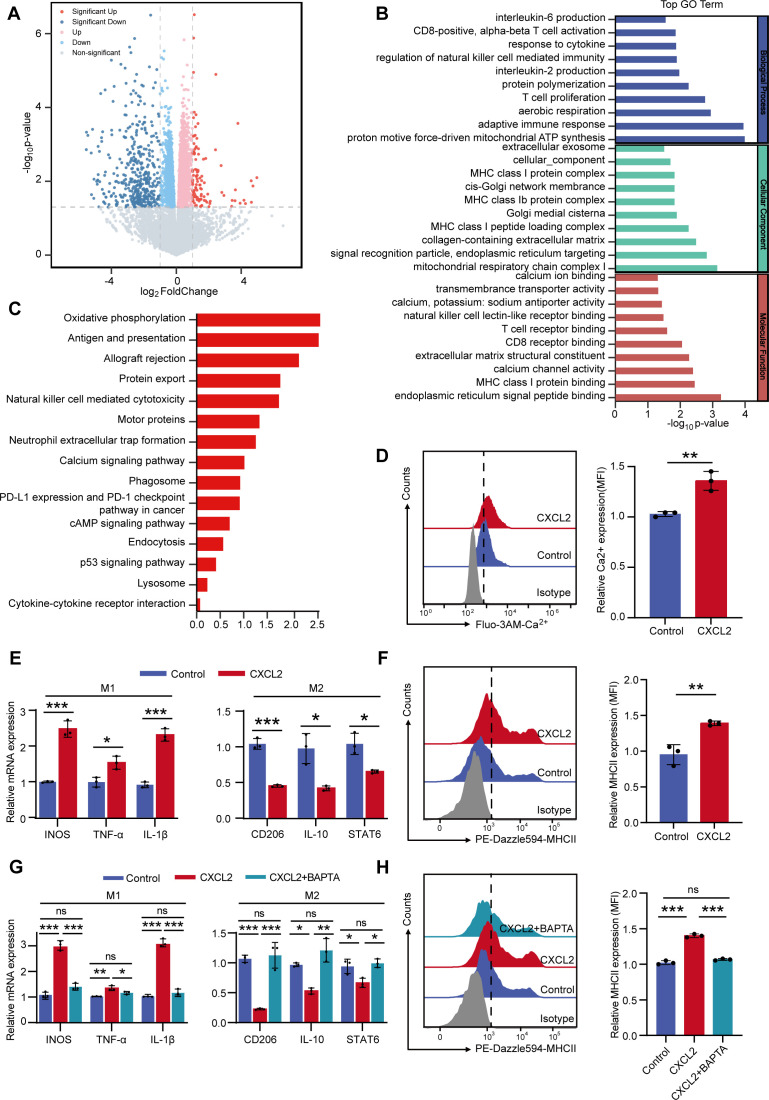
CXCL2 regulated the transition of macrophages to an immune-activated state by mediating cytoplasmic calcium influx. **(A)** Volcano plot of DEGs between DMSO and CXCL2 treatment groups. **(B, C)** GO and KEGG analysis of DEGs between DMSO and CXCL2 treatment groups. **(D)** Flow cytometric analysis of fluo‐3AM positive BMDMs following DMSO and CXCL2 treatment groups. **(E)** qPCR detecting the indicated genes expression levels on BMDMs in DMSO and CXCL2 treatment groups. **(F)** Flow cytometry analysis of MHC-II expression on BMDMs in DMSO and CXCL2 treatment groups. **(G)** qPCR detecting the indicated genes expression levels on BMDMs in the indicated groups. **(H)** Flow cytometry analysis of MHC-II expression on BMDMs in the indicated groups. *P < 0.05, **P < 0.01, ***P < 0.001, and NS, not significant; Student’s t-test or one-way ANOVA test. CXCL2, CXC chemokine ligand 2; DMSO, dimethyl sulfoxide; GO, gene ontology; KEGG, Kyoto Encyclopedia of Genes and Genomes; MFI, median fluorescence intensity; ANOVA, analysis of variance; BMDMs, bone marrow-derived macrophages; DEG, differentially expressed gene; MHC, major histocompatibility complex; mRNA, messenger RNA; qPCR, quantitative PCR.

To investigate the mechanism by which CXCL2 modulates macrophage immune status, we performed transcriptome sequencing on BMDMs stimulated with recombinant CXCL2 protein. The analysis revealed 109 significantly upregulated genes and 404 significantly downregulated genes in CXCL2-treated BMDMs compared to controls (**Figure 3A**). GO analysis indicated that these differentially expressed genes were primarily involved in immune cell activation, cytokine production, and ion signaling pathways (**Figure 3B**). KEGG enrichment analysis further highlighted the involvement of calcium ion signaling (**Figure 3C**), which has been reported to regulate macrophage polarization (24). We evaluated the effect of CXCL2 on intracellular Ca²^+^ concentrations by measuring Fluo-3AM fluorescence intensity using flow cytometry. As shown in **Figure 3D**, CXCL2 treatment increased the cytosolic Ca^2+^ concentration in macrophages. qPCR showed elevated transcript levels of INOS, TNF-α, and IL-1β, along with reduced levels of CD206, IL-10, and STAT6, in CXCL2-treated macrophages (**Figure 3E**). Flow cytometry also demonstrated increased MHC-II expression in BMDMs after CXCL2 stimulation (**Figure 3F**). These results confirmed the shift of macrophages toward an immune-activated state after CXCL2 treatment. Based on the results showing that CXCL2 induced the cytosolic Ca^2+^ influx, we verified whether Ca^2+^ directly regulates CXCL2‐induced macrophage polarization. Our findings showed that the calcium chelator BAPTA inhibited CXCL2-mediated macrophage polarization to an immune-activated state (**Figures 3G, H**). These results suggest that CXCL2 promotes calcium influx to regulate macrophage transition to an immune-activated phenotype.

### CXCL2 inhibits tumor growth in the mouse ESCC subcutaneous tumor model

Given the important role of macrophages in the TME, we explored whether CXCL2 could exert anti-tumor effects in mouse ESCC model. We established an AKR-derived ESCC subcutaneous tumor model and treated mice with recombinant CXCL2 protein.

**Figure 4:**
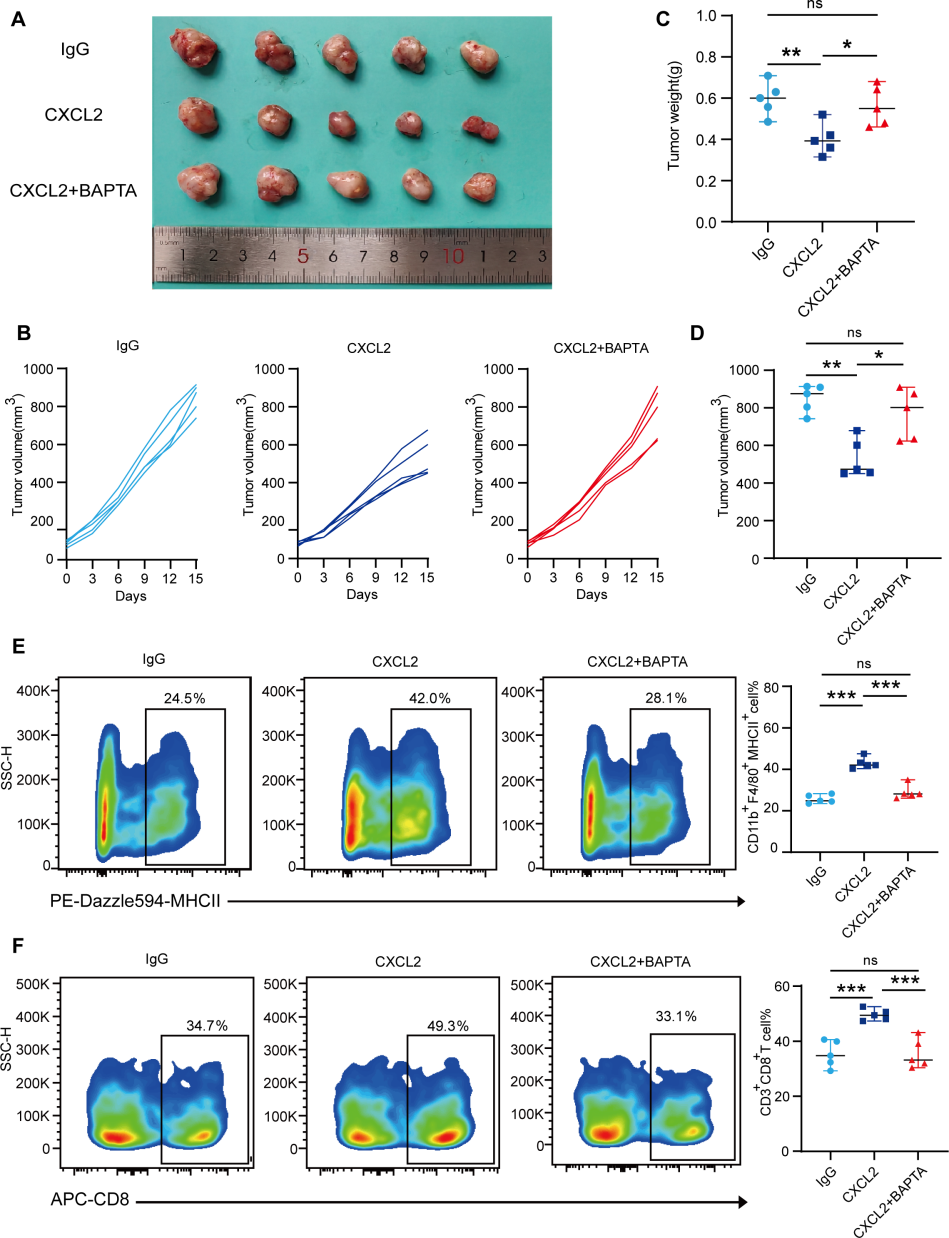
CXCL2 inhibits tumor growth in the mouse ESCC subcutaneous tumor model. **(A)** Gross appearance of subcutaneous ESCC tumors in each treatment group. **(B)** Changes in subcutaneous tumor volumes in each group during the experiment. **(C, D)** Tumor volumes and weights in each group at the end of the experiment. **(E, F)** Flow cytometry analysis depicting the proportion of CD11b^+^F4/80^+^MHCII^+^ macrophages and CD3^+^CD8^+^ T cells in each group. *P < 0.05, **P < 0.01, ***P < 0.001, and NS, not significant; one-way ANOVA test. CXC chemokine ligand 2; ANOVA, analysis of variance; ESCC, esophageal squamous cell carcinoma; MHC, major histocompatibility complex.

We observed that CXCL2 treatment significantly inhibited tumor growth rate, reduced tumor volume, and decreased body weight at the end of the experiment. In contrast, the calcium chelator BAPTA attenuated the tumor growth inhibitory effects of CXCL2 (**Figures 4A–D**). To further elucidate the mechanism underlying the enhanced anti-tumor efficacy of the CXCL2, we analyzed the tumor immune infiltration landscape in tumors. We found that CXCL2 treatment increased the level of CD11b^+^F4/80^+^MHCII^+^ macrophage infiltration, and this effect could be blocked by BAPTA, consistent with our findings *in vitro*. Additionally, CXCL2 treatment enhanced CD3^+^CD8^+^ T cell infiltration in ESCC microenvironment. These results demonstrate that CXCL2 inhibits tumor growth by modulating CD11b^+^F4/80^+^MHCII^+^ macrophage and CD3^+^CD8^+^ T cell infiltration in the mouse ESCC model (**Figures 4E, F**). To validate whether the antitumor function of CXCL2 is macrophage-dependent, we treated subcutaneous tumor model of ESCC with recombinant CXCL2 protein and clodronate liposomes. As shown in **Supplementary Figure 9**, recombinant CXCL2 protein inhibited ESCC growth, and this effect was antagonized in the combined treatment group. Furthermore, flow cytometric analysis revealed an increased proportion of CD11b^+^F4/80^+^ macrophages in ESCC tumors treated with recombinant CXCL2 compared to control group. The combined treatment group exhibited a significantly reduced proportion of CD11b^+^F4/80^+^ macrophages, suggesting that the inhibitory effect of CXCL2 on ESCC tumor growth depend on macrophages.

### CXCL2 enhances the efficacy of anti-PD-1 antibody in ESCC *in vivo*

**Figure 5:**
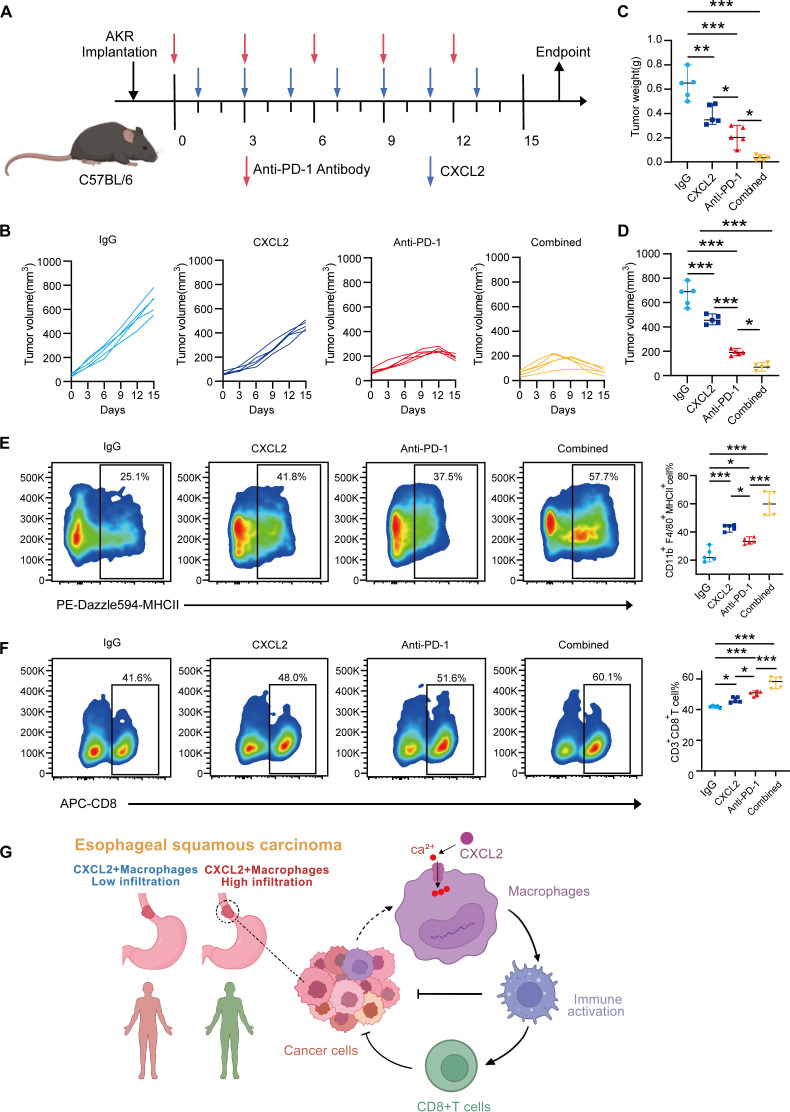
CXCL2 enhances the efficacy of anti-PD-1 antibody in ESCC *in vivo*. **(A)** Schematic of the schedule of anti-PD-1 antibody and CXCL2 treatment in AKR cell-derived subcutaneous ESCC mouse models. **(B)** Changes in subcutaneous tumor volumes in each group during the experiment. **(C, D)** Tumor volumes and weights in each group at the end of the experiment. **(E, F)** Flow cytometry analysis depicting the proportion of CD11b^+^F4/80^+^MHCII^+^ macrophages and CD3^+^CD8^+^ T cells in each group. **(G)** Schematic diagram illustrating the role of CXCL2 in macrophage and the microenvironment immune landscapes of ESCC. Patients with ESCC with immunotherapy responsive typically exhibit a significant infiltration of CXCL2^+^ macrophages, which facilitate the polarization of macrophages to an immune-activated state through calcium influx, thereby enhancing the cytotoxic function of CD8^+^ T cells. *P < 0.05, **P < 0.01, ***P < 0.001, and NS, not significant; one-way ANOVA test. ANOVA, analysis of variance; ESCC, esophageal squamous cell carcinoma; CXC chemokine ligand 2; MHC, major histocompatibility complex; IgG, immunoglobulin G; PD-1, programmed cell death protein-1.

Given the above findings, we investigated whether CXCL2 could enhance the therapeutic efficacy of ESCC immunotherapy. We similarly established an AKR-derived subcutaneous ESCC mouse model and treated them with IgG, CXCL2, anti-PD-1 antibody, or a combination of CXCL2 and anti-PD-1 antibody (**Figure 5A**). During treatment, tumor growth was significantly inhibited in the CXCL2, anti-PD-1, and combination treatment groups (**Figure 5B**). Combination therapy observed the most pronounced anti-tumor effect, which exhibited the smallest tumor volume and lowest tumor weight at the end of the experiment (**Supplementary Figure 2**, **Figures 5C, D**). Flow cytometric analysis revealed that combined therapy significantly increased the infiltration of CD11b^+^F4/80^+^ MHCII^+^ macrophages and CD3^+^CD8^+^ T cells compared to monotherapy and control groups (**Figures 5E, F**). This indicates an improved tumor immune microenvironment. For details on the gating strategy for flow cytometry, see **Supplementary Figure 7**. Furthermore, flow cytometric analysis of mouse ESCC tumor tissues revealed an increased proportion of CD8^+^IFN-γ^+^ cells relative to CD11b^+^F4/80^+^CD206^+^ cells in the CXCL2 treatment group, while the CD11b^+^F4/80^+^CD206^+^ cell proportion decreased. These trends were more pronounced in the combination therapy group (**Supplementary Figure 8**). This suggests that CXCL2 enhances the antitumor function of CD8^+^ T cells while simultaneously suppressing macrophage polarization toward the M2 phenotype. In summary, CXCL2 enhances the efficacy of anti-PD-1 therapy in ESCC by regulating macrophage functional status.

## Discussion

The low response rate to immunotherapy in ESCC patients is primarily attributed to the highly immunosuppressed state of microenvironment, which poses significant challenges for eradicating cancer cells and inducing anti-tumor immunity. Macrophages, as the predominant immune cell population in the TME, exhibit considerable plasticity and heterogeneity (6). Consequently, targeting macrophages to enhance immunotherapeutic efficacy has become a focal point of current research. In this study, we identified a CXCL2^+^ macrophage subpopulation associated with immunotherapy efficacy in the ESCC through scRNA-seq and further elucidated its clinical significance.

Recent studies have highlighted the role of CXCL2 in mediating bidirectional crosstalk between immune cells and tumor cells. For instance, CXCL2 secretion by transformed mesenchymal stromal cells in colorectal cancer promotes M2 macrophage infiltration and tumor cell lung metastasis (25). Conversely, the tumor-suppressive role of CXCL2 was first determined in the study carried out by Ding et al. CXCL2 overexpression takes part in the inhibition of ERK1/2 signaling pathways and promotes apoptosis, therefore suppressing hepatocellular carcinoma development (26). Through single-cell sequencing analysis, researchers identified a novel macrophage subcluster, Mac-cC1QC, which favors an M1 phenotype and exhibits elevated expression of inflammatory cytokines, including CXCL1 and CXCL2, playing a beneficial role in NACI (27). These findings suggest that CXCL2 may modulate macrophage polarization under specific conditions, highlighting the need for further studies to comprehensively elucidate its complex roles in diverse disease contexts. The specific role of CXCL2 in the ESCC microenvironment remains unexplored. Our analysis demonstrated higher CXCL2 expression in macrophages from immunotherapy-responsive patients and revealed better OS in ESCC patients with high CXCL2^+^ macrophage infiltration. Additionally, our study demonstrated that exogenous CXCL2 administration inhibits tumor growth and enhances the infiltration of MHC-II^+^ macrophages and CD8^+^ T cells, improving the tumor immune microenvironment. Furthermore, single-cell sequencing revealed high CXCL2 expression in monocytes, suggesting that in-depth studies on the effects of CXCL2 on various immune cells and their underlying mechanisms could facilitate the development of precise targeted therapies. Besides, single-cell sequencing data from the independent cohort GSE160269 revealed that CXCL2 is not only predominantly derived from monocytes and macrophages but also significantly expressed in fibroblasts and endothelial cells. Although this study focuses on the role of macrophage-derived CXCL2 in ESCC, the contribution of fibroblast-derived CXCL2 warrants further investigation.

Intracellular Ca²^+^ is a critical divalent cation that regulates diverse cellular functions, including cell plasticity, proliferation, and differentiation (28). It has been shown to modulate macrophage polarization: elevated intracellular Ca²^+^ levels enhance macrophage immune function, while reduced levels impair phagocytosis and inflammatory mediator production (24, 29). Our *in vitro* experiments demonstrated that CXCL2 treatment significantly increases intracellular Ca²^+^ influx in macrophages, promoting a shift toward an anti-tumor phenotype. This effect was reversed by the Ca²^+^ chelator BAPTA-AM, which inhibited CXCL2-mediated immune activation. Cytosolic Ca²^+^ changes primarily result from the release of Ca²^+^ from endoplasmic reticulum (ER) stores and the influx through plasma membrane Ca²^+^ channels, which constitute store-operated Ca²^+^ entry (SOCE) (30). Although our findings demonstrate that CXCL2 enhances cytoplasmic Ca²^+^ influx and promotes immune activation, further studies are required to elucidate the mechanisms underlying Ca²^+^ release from the ER and influx through the plasma membrane. A growing number of studies have focused mainly on the role of Ca^2+^ release-activated Ca^2+^ (CRAC) channels in immune cell function, but their role in macrophage responses and immune regulation remains poorly understood (31). Hui Dong et al. demonstrated that CaSR and TRPV4 channels promote Ca²^+^-dependent M1 macrophage polarization through a novel coupling of the PLA2/CYP450 and PLC/PKC pathways (24). During CXCL2-stimulated M1 macrophage polarization, the specific calcium channels involved, as well as their expression patterns, remain to be elucidated.

Additionally, the limited sample size of scRNA-seq and patient specimens for prognostic analysis necessitates further validation through basic research and large, multicenter patient cohorts in future *in vivo* and *in vitro* experiments.

## Conclusion

In conclusion, our study revealed the high expression of CXCL2 in macrophages in ESCC and identified the level of CXCL2^+^ macrophage infiltration as an independent factor for OS in ESCC. CXCL2^+^ macrophages exhibited a highly immune-activated phenotype, and CXCL2 enhanced anti-PD-1 efficacy of ESCC by modulating macrophage functional status (**Figure 5G**).

## Data Availability

The original contributions presented in the study are included in the article/**Supplementary Material**. Further inquiries can be directed to the corresponding authors.
