# Supplementary material for: CXCL2 affects macrophage antitumor function and immunotherapy efficacy in esophageal squamous cell carcinoma through calcium signaling
Source: Front Immunol. 2026 Apr 13;17:1695387. doi: 10.3389/fimmu.2026.1695387 (PMC13111272; doi:10.3389/fimmu.2026.1695387)
Supplement: Supplementary file 1 [file DataSheet1.pdf]

Supplemental Data

CXCL2^{+} macrophages determine the immune- activated microenvironment and efficacy of immunotherapy in esophageal squamous cell carcinoma

The Supplemental Data consist of:

Supplemental Figures 1-9

Supplemental tables 1-2

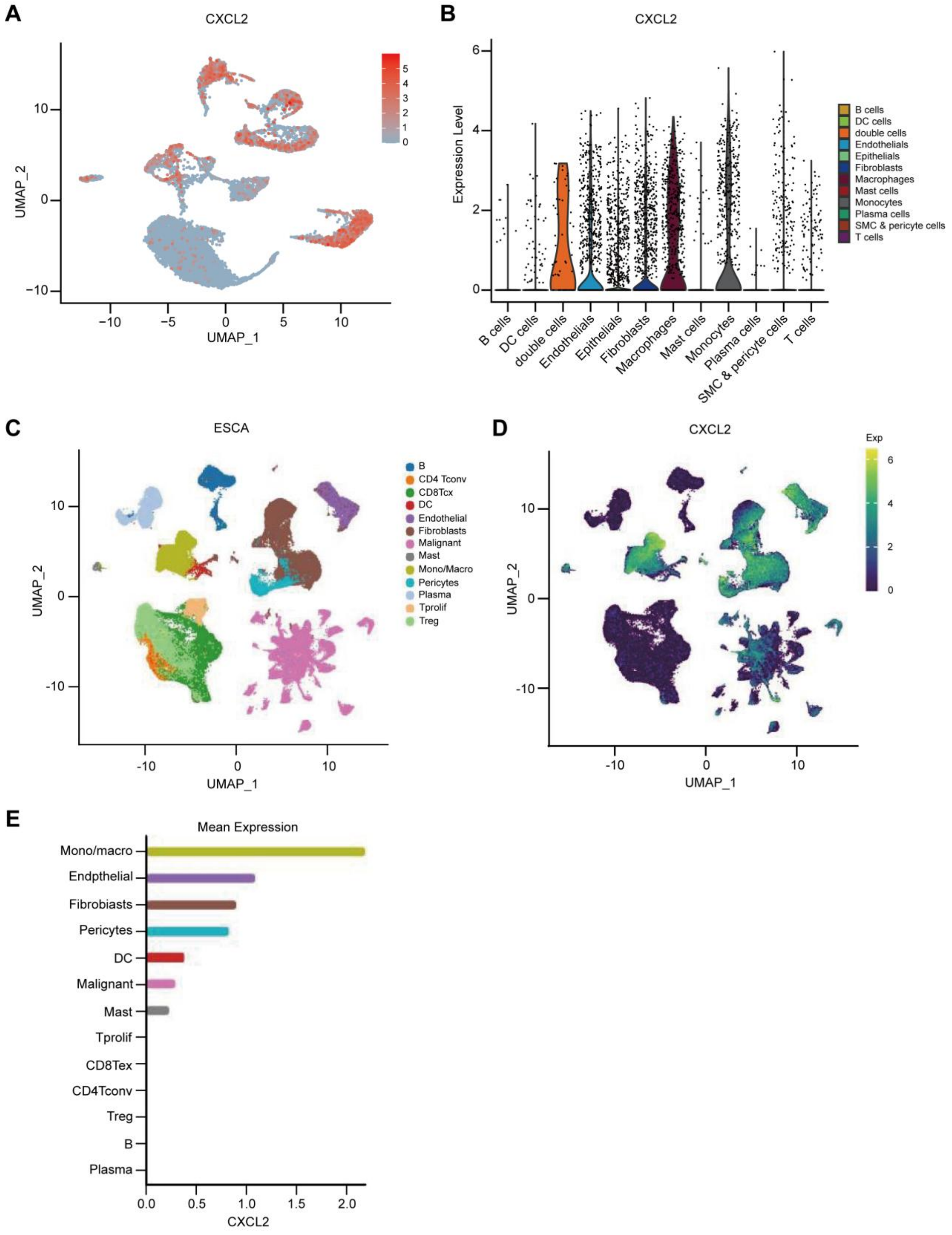

Supplemental Figure 1. (A) UMAP plot of cell clusters colored by the expression level of CXCL2. (B) Violin plot of the CXCL2 expression level in each cell cluster. (C) UMAP plot of single cells from patients with ESCA in GSE160269 cohort. (D) UMAP plot of cell clusters colored by the expression level of CXCL2 in GSE160269. (E) Expression level of CXCL2 on different cells in GSE160269.

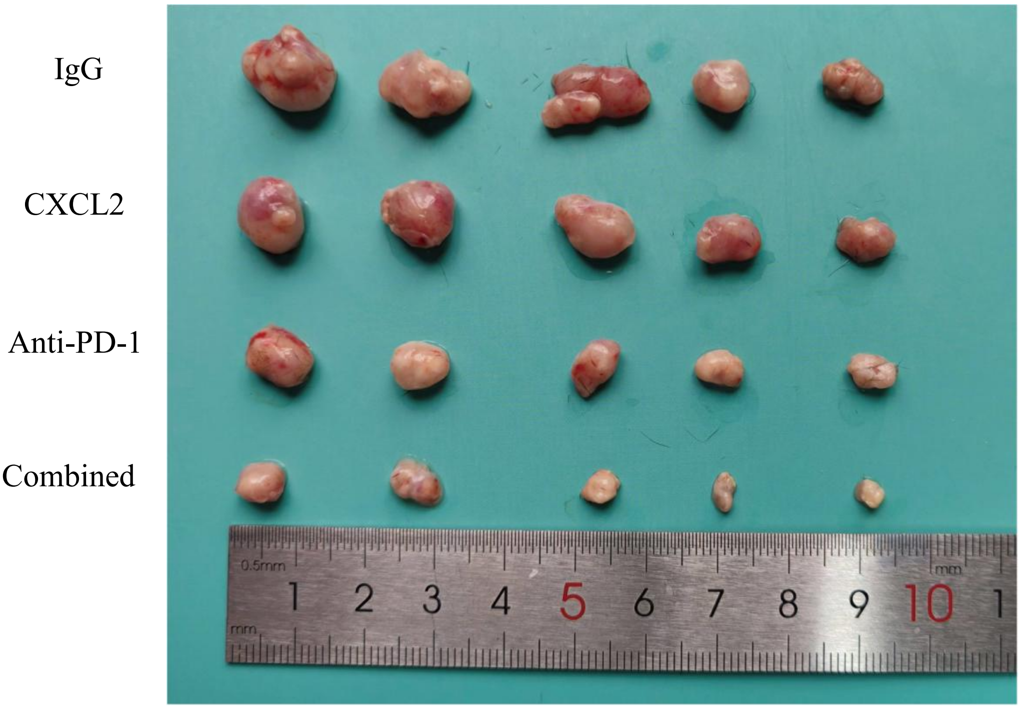

Supplemental Figure 2. Gross appearance of subcutaneous ESCC tumors in each treatment group.

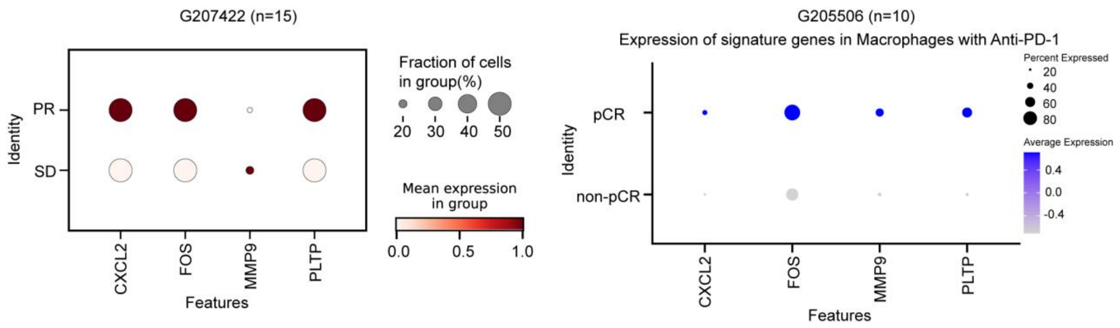

Supplementary Figure 3. The expression levels of CXCL2 in immunotherapy responders and non-responders in GSE207422 and GSE205506.

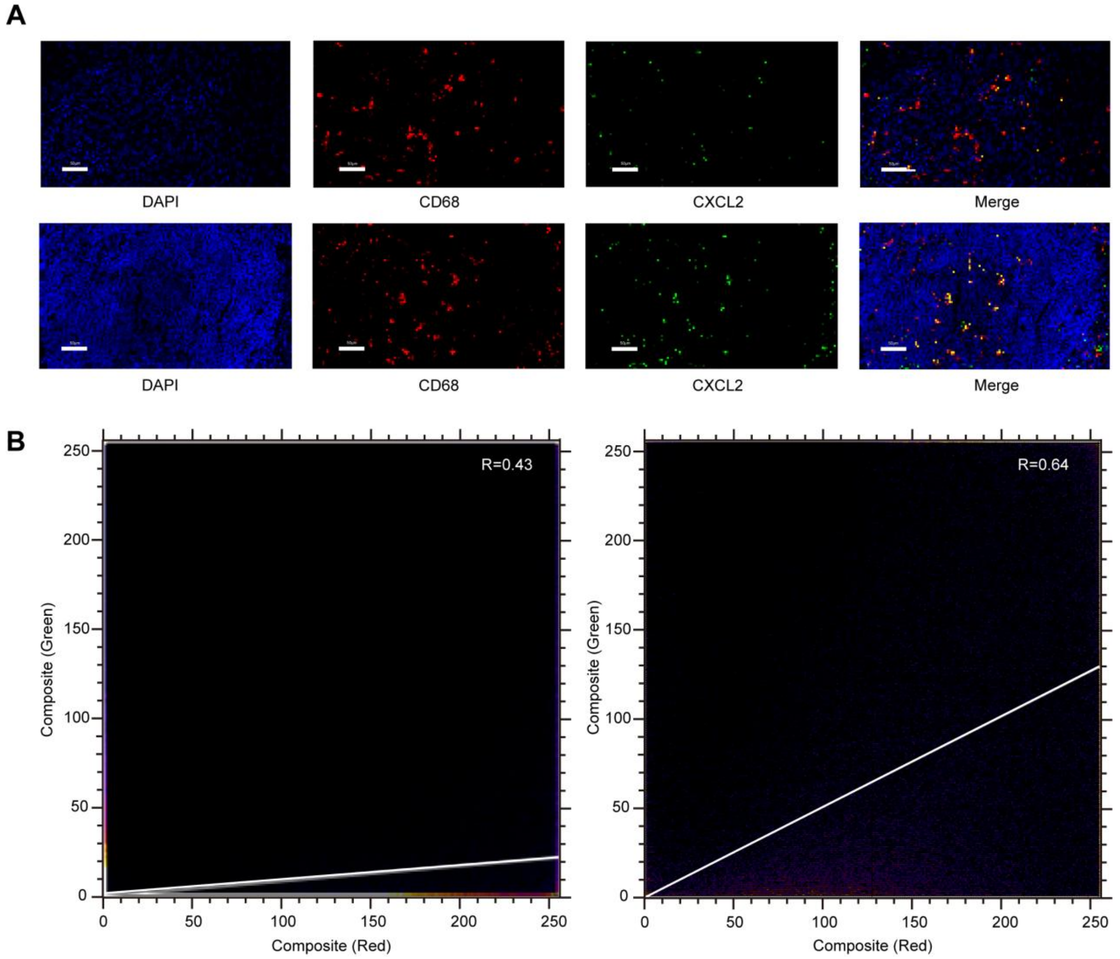

Supplementary Figure 4. Co-localization of CXCL2 and CD68 in different regions of ESCC tumors. (A) Representative images of immunofluorescence co-staining for CD68 (red) and CXCL2 (green) in the tumor extremity region (top) and tumor core region (bottom). Scale bar, 50 μm. (B) Correlation coefficient of CXCL2 and CD68 fluorescence intensity in in the tumor extremity region (left) and tumor core region (right).

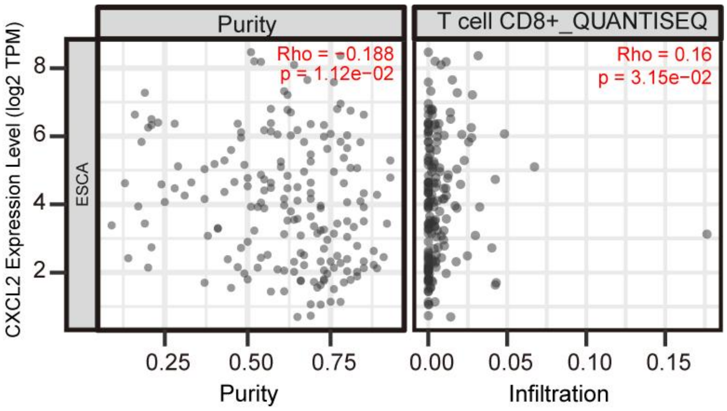

Supplementary Figure 5. The correlation analysis of CXCL2 expression level with the infiltration proportion of $CD8^+$ T cells in ESCC.

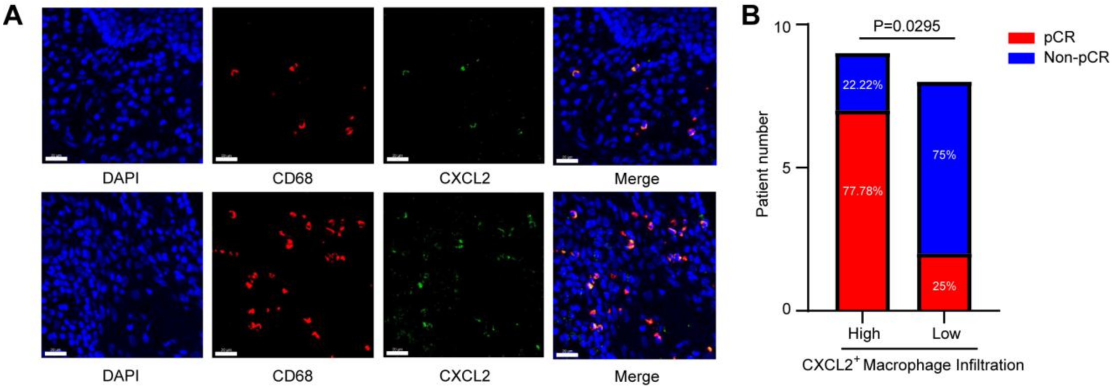

Supplementary Figure 6. $CXCL2^+$ macrophage infiltration correlates with immunotherapy response in ESCC. (A) Representative images of immunofluorescence co-staining for CD68 (red) and CXCL2 (green) in tumor sections from pCR (bottom) and non-pCR (top) patients. Scale bar, 20 µm. (B) The percentage of non-pCR and pCR patients following immunotherapy with high and low $CXCL2^+$ macrophage infiltration.

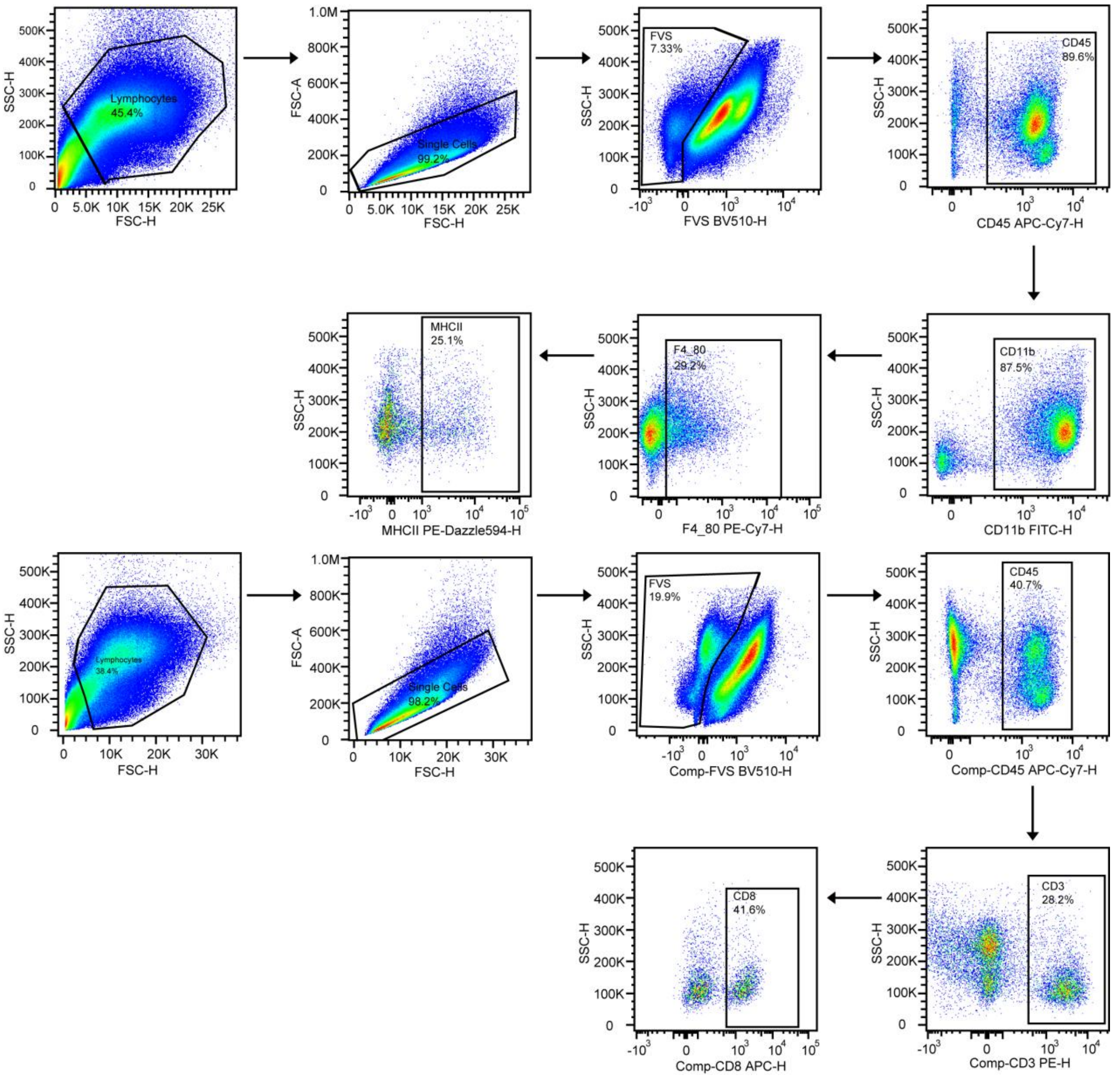

Supplementary Figure 7. Representative example of the gating strategy of MHCII^{+} macrophage and CD8^{+} T cells.

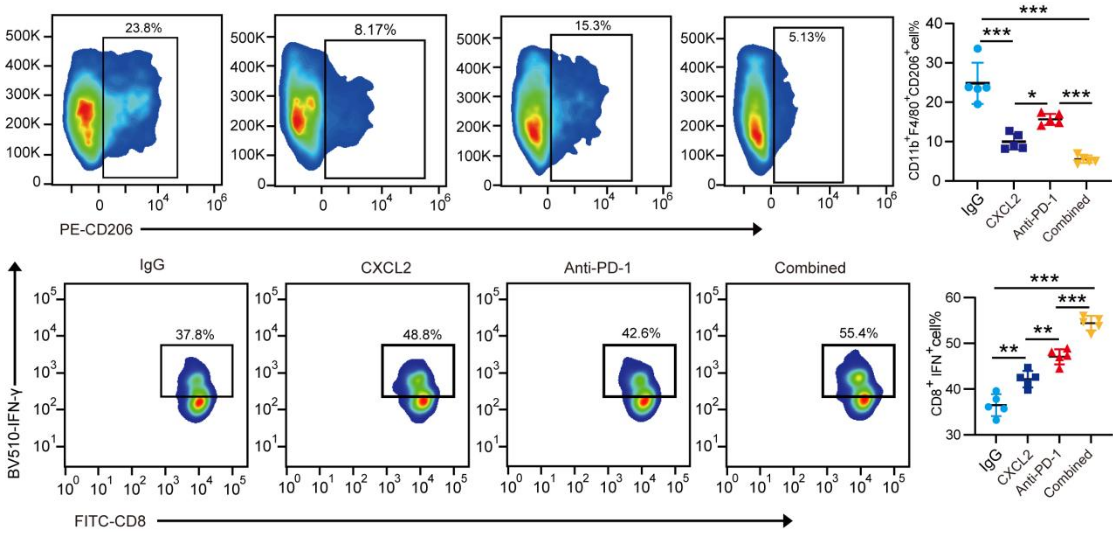

Supplementary Figure 8. Flow cytometry analysis revealed the proportion of CD11b^{+}F4/80^{+}CD206^{+} cells and CD8^{+}IFN^{+} cells in each group. * $P < 0.05$, ** $P < 0.01$, *** $P < 0.001$.

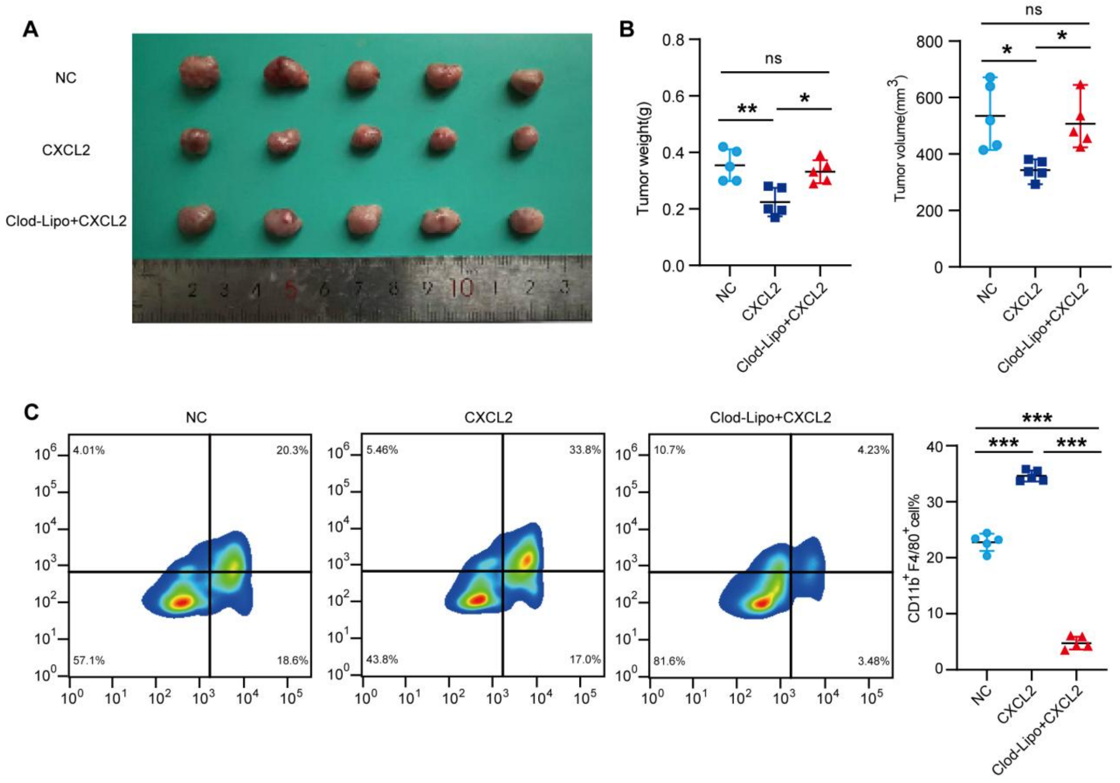

Supplementary Figure 9. CXCL2 exerts antitumor effects in a macrophage-dependent manner in ESCC. (A) Gross appearance of subcutaneous ESCC tumors in each treatment group. (B)Tumor volumes and weights in each group at the end of the experiment. (C) Flow cytometry analysis depicting the proportion of CD11b^{+}F4/80^{+}macrophages in each group. * $P<0.05$, ** $P<0.01$, *** $P<0.001$.

Supplementary Table 1. The clinicopathologic details of ESCC patients (n=74)

Variables	Number
Age(years)	
≤ 50	25 (33.78%)
> 50	49 (66.21%)
Gender	
Female	5 (6.75%)
Male	69 (93.25%)
TNM stage	
I+II	27 (36.49%)
III+IV	47 (63.51%)
Tumor differentiation	
G1+G2	40 (54.05%)
G3+G4	34 (45.95%)
Treatment	
Surgery	66 (89.2%)
Chemotherapy	8 (10.8%)

Supplemental Table 2. Sequences of primers (5'-3') used for qRT-PCR.

Genes		Sequences (5'-3')
Mouse CD206	FORWARD	CTCTGTTCAGCTATTGGACGC
Mouse CD206	REVERSE	TGGCACTCCCAAACATAATTTGA
Mouse STAT6	FORWARD	CATCTGAACCGACCAGGAACT
Mouse STAT6	REVERSE	CTCTGTTCAGCTATTGGACGC
Mouse GAPDH	FORWARD	AGGTCGGTGTGAACGGATTTG
Mouse GAPDH	REVERSE	TGTAGACCATGTAGTTGAGGTCA
Mouse IL-1 β	FORWARD	GGCCAGATCCTGTCCAAGC
Mouse IL-1 β	REVERSE	TGGATGCTCTCATCAGGACAG
Mouse IL-10	FORWARD	CGCAGCTCTAGGAGCATGTG
Mouse IL-10	REVERSE	TGTTTCCGTGGAGACGCAAG
Mouse TNF-a	FORWARD	CAGGCGGTGCCTATGTCTC
Mouse TNF-a	REVERSE	CGATCACCCCGAAGTTCAGTAG
Mouse INOS	FORWARD	GTTCTCAGCCCAACAATACAAGA
Mouse INOS	REVERSE	GTGGACGGGTCGATGTCAC
